# Exploring the influence of the spiritual climate on psychological empowerment among nurses in China: a cross-sectional study

**DOI:** 10.1186/s12912-024-02011-x

**Published:** 2024-06-03

**Authors:** Xuan Wang, Yulan Xia, Li Gou, Xianxiu Wen

**Affiliations:** 1 Department of Nursing, Sichuan Provincial People’s Hospital, school of medicine, University of Electronic Science and Technology of China, 32 West second Section, 1st Ring Road, Chengdu, 610072 China; 2 Department of Nursing, Sichuan Provincial People’s Hospital, school of medicine, University of Electronic Science and Technology of China, Chengdu, China; 3 Department of Geriatrics Cardiovascular, Sichuan Provincial People’s Hospital, school of medicine, University of Electronic Science and Technology of China, Chengdu, China; 4 Department of Nursing Research Centre, Sichuan Provincial People’s Hospital, school of medicine, University of Electronic Science and Technology of China, Chengdu, China

**Keywords:** Nurses, Spiritual climate, Psychological empowerment

## Abstract

**Background:**

Psychological empowerment notably impacts nurses’ work engagement and high-quality care. A spiritual climate is a work environment that respects individuals and encourages them to express personal views. Previous studies have shown that a spiritual climate enhances psychological empowerment, however, the relationship between them among the nursing population remains unclear. This study aimed to explore the effect of a spiritual climate on nurses’ psychological empowerment and provide a scientific basis for improving psychological empowerment among nurses.

**Methods:**

A cross-sectional survey of 837 nurses from five hospitals in Sichuan Province, Southwest China, was conducted using a convenience sampling method; this survey included nurses’ demographic characteristics, the Psychological Empowerment Scale (PES), and the Chinese version of the Spiritual Climate Scale (C-SCS). The data were analysed using one-way analysis of variance (ANOVA), correlation analysis, and multiple linear regression.

**Results:**

The sample of 837 nurses attained a psychological empowerment score of (45.49 ± 6.42) and a spiritual climate score of (75.25 ± 16.75). The one-way ANOVA revealed that psychological empowerment scores among nurses varied based on differences in age, department, years of work experience, professional title, level of work intensity, and children (yes/no). *Pearson’s* correlation analyses revealed a significant positive correlation between the spiritual climate and nurses’ psychological empowerment (*r* = 0.564, *P* < 0.001), and multiple linear regression analysis showed that working in the intensive care unit (ICU), work intensity, and the four items pertaining to spiritual climate influenced nurses’ psychological empowerment, explaining 32.6% of the total variance in psychological empowerment.

**Conclusion:**

The findings suggested that the spiritual climate perceived by nurses and psychological empowerment are moderately high. Working in the ICU, work intensity, and the four items pertaining to spiritual climate influence nurses’ psychological empowerment. Nursing managers should pay attention to the daily work intensity of nurses, especially ICU nurses, organize work tasks reasonably, promote dynamic and balanced nurse human resource deployment based on patients’ conditions and nurses’ workloads, and implement scientific scheduling plans to establish a positive spiritual climate in the workplace. Additionally, group workshops and systematic training programs can effectively enhance psychological empowerment among nurses.

## Background

Nurses are the largest group of actors in the healthcare system and play an essential role in maintaining human health, ensuring patient safety, and promoting the development and progress of healthcare [1, 2]. Especially in the wake of the COVID-19 pandemic, nurses have received widespread attention as the cornerstone of quality health services, and positive perceptions of nurses on the part of global citizens are evident [3]. These perceptions demand a higher level of professional service competence on the part of nurses. However, international nurse groups face the issues of human resource shortages [4], heavy workloads [5], and complex clinical environments [6], which have led to increased rates of nurse burnout, reduced job dedication, and poorer self-rated health following the COVID-19 pandemic [7]. These factors have severely impacted the motivation of nurses to provide good care to their patients. Psychological empowerment refers to the psychological experience of an individual during the process of empowerment and reflects that individual’s psychological cognition of the importance of self-worth at work [8]. Nurses with high levels of psychological empowerment exhibited increased levels of work engagement and can provide high-quality care to patients [9, 10]. A spiritual climate is a work environment in which individuals and team members cooperate and interact with each other in the workplace, thereby establishing trusting relationships and sharing inner experiences [11]. The significant predictive effect of a spiritual climate on psychological empowerment has been demonstrated in lecturers at higher education institutions in the field of tourism and hospitality [12]. To our knowledge, the relationship between these factors among nurses has not been explored; therefore, our study attempts to fill this gap in the literature.

Spreizer defined psychological empowerment as the cognitive feeling of being empowered, which is experienced by an individual in a particular work situation and takes the form of measurable perceptions that reflect that individual’s active orientation towards his or her work role [13]. In this study, psychological empowerment refers to the empowerment that nurses feel at work due to their supervisors or organizations, and it reflects the positive attitudes towards personal growth and development that nurses experience as part of their work roles. The formulation of Spreizer’s theory of psychological empowerment laid the foundation for the development of empirical research on this notion in various disciplines. The positive role of psychological empowerment in nursing management has continued to be confirmed as research on this topic has advanced. Psychological empowerment can enhance nurses’ work engagement [10]. Nurses who exhibit a higher level of psychological empowerment are better able to recognize the significance of nursing and to work more autonomously as a result of their skills and knowledge. Li et al. [14] revealed a significant correlation between psychological empowerment and job satisfaction; namely, nurses who perceive more psychological empowerment exhibit increased job satisfaction and enhanced retention. In addition, psychological empowerment reduces nurses’ sense of occupational stress [15] and burnout [16], which is necessary for their ability to provide high-quality care to patients [9].

Given the important role of psychological empowerment, it is particularly valuable to conduct an active exploration of the factors influencing psychological empowerment and to seek measures to improve psychological empowerment among nurses. Several sociodemographic factors [17], such as gender, age, years of work experience, job title, and level of education, have been suggested to influence nurses’ psychological empowerment; however, the results of studies of the effects of demographic characteristics on nurses’ psychological empowerment have been mixed. Khoshmehr et al. found no significant effect of gender or level of education on nurses’ psychological empowerment [18], but another study reported that these factors were associated with psychological empowerment [19]. These findings suggest that the influences of psychological empowerment require further exploration and provide direction for selecting such potential influences.

The notion of a spiritual climate refers to the shared perceptions of employees towards spirituality and both the state and process of sharing workplace spirituality [20]. An increasing number of researchers have started to pay attention to and investigate workplace spirituality, and the question of how to establish a positive spiritual climate in the workplace has gradually become a research hotspot in various disciplines. In the field of healthcare, Doram [11] defined a spiritual climate as a work environment that respects and encourages individuals to express their views and guides individuals to play their roles and devote themselves wholeheartedly to their work. A study conducted in China verified the mediating role of spiritual climate in the relationship between burnout and turnover intention, revealing that nurses in a positive spiritual climate were less likely to experience burnout or thoughts about leaving [21]. Moreover, studies have shown that a spiritual climate at work enhances employees’ well-being, work engagement, caring behaviour, and job satisfaction [22–25]. Work meaning is one of the fundamental aspects of psychological empowerment [13]. Jufrizen et al. [26] suggested that the spiritual climate can help deepen an individual’s cognition of the meaning of his or her work, and a positive spiritual climate has also been shown to increase nurses’ work meaning [27]. In addition, studies have confirmed that work environment factors influence nurses’ psychological empowerment and that an excellent organizational climate is conducive to the enhancement of nurses’ level of psychological empowerment [28, 29]. These findings provide a theoretical basis for understanding the impact of spiritual climate on psychological empowerment.

According to the conceptual analysis and an interpretive model of empowerment developed by Thomas and Velthouse [30], empowerment involves a set of perceptions that are shaped by the work environment. The spiritual climate is a superior work environment that can enhance workers’ relationships with their coworkers, thus enabling them to find meaning and purpose in their work. In other words, a spiritual climate at work is likely to activate individual psychological empowerment. Based on this theoretical framework and the literature review presented above, it is reasonable to assume that a spiritual climate may be related to nurses’ psychological empowerment. However, despite the fact that previous studies have highlighted the conceptual link between spiritual climate and psychological empowerment, no empirical studies have explicitly explored the relationship between these factors among nurses. Therefore, this study analyses the level of psychological empowerment exhibited by nurses in China through a cross-sectional survey, constructed a multiple linear regression analysis model, and used the least squares method (LSE) to assess the parameters and explores the influence of spiritual climate on psychological empowerment, thereby not only expanding the research on the influence of a spiritual climate but also providing a scientific basis for improving the climate of nurses’ work environment and increasing their level of psychological empowerment.

## Methods

### Study design, participants, and setting

Sichuan Province is located in the southwestern interior of China and consists of five regions: East Sichuan, South Sichuan, West Sichuan, North Sichuan, and Central Sichuan. In this study, due to geographical constraints, a questionnaire survey was conducted from February to May 2023 using the convenience sampling method; this survey focused on registered nurses working in five hospitals in these five regions, all of which were tertiary care public hospitals featuring more than 1,500 beds. The study inclusion criteria for nurses focused on (1) registered nurses who (2) had at least one year of nursing experience in their current hospital and (3) agreed to participate voluntarily in this study. The exclusion criteria focused on nurses who were (1) not directly involved in patient care, such as those who were on sick or personal leave, and (2) nursing managers at the level of the head nurse or above. According to the sample size estimation requirements for multiple linear regression analysis studies, the sample size for the study was calculated using an estimation method that requires the sample size to be ten to twenty times the number of questionnaire items [31]. This study ultimately included 26 items, of which 10 items focused on nurses’ general information, 4 items were drawn from the spiritual climate scale and 12 items were drawn from the psychological empowerment scale; accordingly, *N* = (10 + 4 + 12) *20 = 520. To take the possibility of invalid questionnaires into account, the sample size was expanded by 20%, and the final sample size estimated for this study was at least 650. Ultimately, the sample size was 837, which met the sample size requirement.

### Measurements

#### General information questionnaire

The research team designed a questionnaire to collect the general information of nurses based on a literature review and expert consultations. This questionnaire included a total of 10 items pertaining to nurses’ gender, age, department, years of work experience, level of education, professional title, employment type, work intensity, marital status, and children (yes/no).

#### Psychological empowerment scale

The Psychological Empowerment Scale (PES), which is used to measure psychological empowerment, was developed by Spreitzer et al. [13] and subsequently translated into Chinese and validated by Sun et al. [32]. The scale contains 12 items across four subscales, including job meaning (3 items), job competence (3 items), self-efficacy (3 items), and job impact (3 items). An example item is “I am confident in my ability to do well in all aspects of my job.” Each item is measured on a 5-point Likert scale ranging from “strongly disagree = 1” to “strongly agree = 5.” Total scores on the scale range from 12 to 60. High scores indicate high perceived psychological empowerment on the part of nurses. The Cronbach’s alpha coefficients for the four subscales of the original scale range from 0.72 to 0.86. Before the formal commencement of the investigation in this study, a pilot study was conducted by reference to 30 registered nurses who met the inclusion criteria. The Cronbach’s alpha coefficients for the four dimensions of the PES in the pilot study ranged from 0.80 to 0.95, as calculated by using SPSS 27.0 software for analyses; ultimately, the Cronbach’s alpha coefficients for the present study ranged from 0.83 to 0.94.

#### Spiritual climate scale

Wu et al. developed and revised the Chinese version of the Spiritual Climate Scale [33] based on Doram et al. [11]. The C-SCS is a single-dimensional scale featuring four items. Item 1 is “My department encourages and supports the opinions and ideas that I propose, and colleagues can listen and be receptive.” Item 2 is " Doctors and nurses respect the opinions and ideas I propose.” Item 3 is “I feel a sense of belonging and identity when I communicate and share my ideas with my colleagues.” Item 4 is “I’m in a department where everyone can express themselves and where we respect and understand each other.” This measure is scored on a 5-point Likert scale ranging from “strongly disagree = 1” to “strongly agree = 5.” The total mean scores for the four items were subtracted from 1 and multiplied by 25 to obtain the final score, which ranged from 0 to 100; the higher the total score was, the better the nurses’ perceived spiritual climate in the workplace. The Cronbach’s alpha coefficient of the scale was 0.83. Furthermore, in the pilot study, the Cronbach’s alpha coefficient was 0.95; ultimately, in this study, the Cronbach’s alpha coefficient of the C-SCS was 0.93.

### Data collection

This study used a paper-based questionnaire to collect information. A member of the research team who served as a head nurse contacted the director of the nursing department of the hospital under investigation, and after providing consent, the nursing department of the hospital in question arranged for a full-time person in charge to interact with the members of the research team. The members of the group contacted the full-time person in charge of each hospital to determine a time when the questionnaire could be distributed, and during the process of formally distributing the questionnaire, the members of the group visited each department of the surveyed hospitals to provide training in questionnaire completion, in which context they explained in detail the precautions to be taken while completing the questionnaire, such as the time required to complete the questionnaire, standardized methods for completing the questionnaire and the importance of completeness. Once the questionnaires were collected, the researcher excluded questionnaires in which general information was lacking, those that featured illogical entry options, and those that were characterized by omissions accounting for more than 10% of the total. The questionnaires were completed during the morning shift or at a group meeting in each department, which included Internal Medicine, Surgery, Obstetrics and Gynaecology, Paediatrics, Emergency, and the Intensive Care Unit (ICU). During the completion of the questionnaire, the researcher was available to provide timely clarification to respondents who did not understand the questions. The questionnaire included the general information questionnaire for nurses, the PES, and the C-SCS. After the data collection concluded, two people verified, organized, and inputted the data. In total, 851 nurses completed and returned questionnaires, of which 14 questionnaires were omitted from the analysis due to incompleteness; thus, 837 valid questionnaires were ultimately analysed (response rate: 98.35%).

### Ethical consideration

The study was approved by the Ethics Committee of the Sichuan Academy of Medical Sciences, Sichuan Provincial People’s Hospital (Approval Number: 202350), i.e., the first author’s institution. Researchers distributed the questionnaire to investigators only after receiving informed consent from each participant. To protect the privacy of the participants, the questionnaires were completed anonymously and retrieved by the researcher as soon as they were completed, thus ensuring that third parties could not see the information that the participants had provided. The paper version of the questionnaire was also treated confidentially in this study; specifically, each questionnaire was coded using numbers that did not identify the participant, and all questionnaires were kept in the researcher’s office for locking to restrict access. In addition, participants were promised that the data collected would be used solely for current academic research. The datasets used and analysed as part of the current study were made available only by emailing the corresponding author and could not be accessed by other parties.

### Data analysis

SPSS 27.0 was used to support the data analysis. All data in this study were normally distributed, and the means ± standard deviations (SDs) were used to describe the variables, while frequencies and percentages were used to describe the count data. Comparisons among groups were performed using independent samples, *t* tests, or analysis of variance (ANOVA). *Pearson’s* correlation was used to analyse the correlation between mental climate and nurses’ psychological empowerment, and multiple linear regression was used to examine the factors influencing psychological empowerment. Differences were two-sided, and *P* < 0.05 was considered to indicate statistical significance.

## Results

### General information regarding nurses and the one-way ANOVA for psychological empowerment

Among the 837 nurses who participated in this survey, 94.1% were female, 49.6% were aged 31 ∼ 40 years, 31.7% worked in Internal Medicine, 55.2% had 1 ∼ 10 years of work experience, 80.9% had a bachelor’s degree, 82.3% were working on contract, and most were married and had children. In addition, a one-way ANOVA revealed statistically significant differences in nurses’ psychological empowerment scores according to their age, department, years of work experience, professional title, work intensity, and children (yes/no); in other words, older nurses, those working in the ICU, those with more years of work experience, those with higher professional titles, those facing lower work intensity, and those with children attained relatively high psychological empowerment scores. The detailed characteristics concerning the remaining demographic details are shown in Table 1.

**Table 1:** General information regarding nurses and the results of a one-way ANOVA on psychological empowerment (*n* = 837)

Item	*n*(%)	Psychological Empowerment		
		Mean ± SD	*t* or *F*	*P*
Gender			-0.919	0.358
Male	49(5.9)	44.67 ± 6.32		
Female	788(94.1)	45.54 ± 6.42		
Age			9.494	<0.001
18 ∼ 30	312(37.3)	45.73 ± 6.12		
31 ∼ 40	415(49.6)	44.74 ± 6.79		
> 40	110(36.6)	47.65 ± 5.20		
Department			5.173	<0.001
Internal Medicine	265(31.7)	46.51 ± 6.59		
Surgery	183(21.9)	45.51 ± 6.16		
Obstetrics and Gynaecology	31(3.7)	45.00 ± 6.86		
Paediatrics	78(9.3)	44.56 ± 6.29		
Emergency	60(7.2)	44.40 ± 5.78		
ICU	87(10.4)	42.66 ± 4.61		
Other	133(15.9)	46.43 ± 7.06		
Years of work experience			3.419	0.033
1 ∼ 10	462(55.2)	45.40 ± 6.46		
11 ∼ 20	287(34.3)	45.13 ± 6.77		
> 20	88(10.5)	47.14 ± 4.57		
Level of education			0.393	0.675
Junior college	138(16.5)	45.71 ± 5.70		
Bachelor’s degree	677(80.9)	45.48 ± 6.57		
Master’s degree or above	22(2.6)	44.41 ± 6.00		
Employment type			-0.259	0.796
Contracted	689(82.3)	45.46 ± 6.50		
Formal	148(17.7)	45.61 ± 6.06		
Professional title			4.244	0.015
Junior	459(54.8)	45.53 ± 6.70		
Middle	319(38.1)	45.03 ± 6.12		
Senior	59(7.0)	47.66 ± 5.24		
Work intensity				<0.001
Low	134(16.0)	47.39 ± 6.35		
Medium	397(47.4)	46.00 ± 6.01		
High	306(36.6)	44.01 ± 6.66		
Marital status			-1.104	0.270
Single	226(27.0)	45.09 ± 6.65		
Married	611(73.0)	45.63 ± 6.33		
Children			2.121	0.034
Yes	531(63.4)	45.85 ± 6.23		
No	306(36.6)	44.88 ± 6.70		

### Psychological empowerment and spiritual climate scores

The total scores for psychological empowerment and spiritual climate among nurses in Sichuan Province, Southwest China, were 45.49 ± 6.42 and 75.25 ± 16.75, i.e., mainly moderate to high, thus indicating a high level of psychological empowerment and perceptions of a favourable spiritual climate in the workplace; these findings are in line with our expected outcomes, as detailed in Table 2.

**Table 2:** Scores on the psychological empowerment scale and the spiritual climate scale (*n* = 837)

Variable	Mean	SD
Psychological empowerment scale	45.49	6.42
Job meaning	12.52	2.00
Job competence	12.68	1.67
Self-efficacy	12.04	1.96
Job impact	8.24	3.00
Spiritual climate scale	75.25	16.75

### Correlation analysis of spiritual climate and psychological empowerment

*Pearson’s* correlation analysis revealed a positive correlation between spiritual climate and psychological empowerment as well as its dimensions (*P* < 0.01), as detailed in Table 3.

**Table 3:** Correlation analysis of spiritual climate and psychological empowerment (*n* = 837)

Variables	1	2	3	4	5	6
1. Psychological empowerment	1					
2. Job meaning	0.727**	1				
3. Job competence	0.746**	0.601**	1			
4. Self-efficacy	0.778**	0.449**	0.593**	1		
5. Job impact	0.732**	0.265**	0.253**	0.381**	1	
6. Spiritual climate	0.564**	0.477**	0.425**	0.432**	0.370**	1

**P < 0.01

### Multiple linear regression analysis of psychological empowerment

In this study, multiple linear regression analysis was performed using an input method to explore the factors influencing psychological empowerment among nurses. The psychological empowerment score of nurses was used as the dependent variable. The scores of the four items pertaining to the spiritual climate as well as variables that were statistically significant according to the one-way ANOVA, such as age, department, years of work experience, professional title, work intensity, and children (yes/no), were used as independent variables. The method of independent variable assignment is shown in Table 4. The results of this study showed that the ICU (*β* = -2.073, *P* = 0.002), work intensity (*β* = -0.602, *P* = 0.033), and the four items associated with the spiritual climate scale influenced nurses’ psychological empowerment, explaining a total of 32.6% of the total variance; the results are shown in Table 5.

**Table 4:** The method of independent variable assignment

Independent variable	Assignment
Age	18 ∼ 30 = 1; 31 ∼ 40 = 2; >40 = 3
Department	Using Internal Medicine as a reference, Surgery = (0,1,0,0,0,0,0); Obstetrics and Gynaecology =(0,0,1,0,0,0,0); Paediatrics=(0,0,0,1,0,0,0); Emergency=(0,0,0,0,1,0,0); ICU=(0,0,0,0,0,1,0); Other=(0,0,0,0,0,0,1)
Years of work experience	1 ∼ 10 = 1; 11 ∼ 20 = 2; >20 = 3
Professional title	Junior = 1; Middle = 2; Senior = 3
Work intensity	Low = 1; Medium = 2; High = 3
Children (yes/no)	Yes = 1; No = 2
SC1	Original score input
SC2	Original score input
SC3	Original score input
SC4	Original score input

SC1-SC4, items pertaining to the spiritual climate

**Table 5:** Multiple linear regression analysis of psychological empowerment (*n* = 837)

Variables	*β*	*SE*	*β*′	*t*	*P*	95% CI	
						LLCI	ULCI
Constant	28.042	2.315		12.112	<0.001	23.498	32.587
Age	-0.076	0.506	-0.008	-0.151	0.88	-1.071	0.918
Department							
Surgery	-0.937	0.508	-0.060	-1.845	0.065	-1.934	0.060
Obstetrics and Gynaecology	-0.495	1.007	-0.015	-0.492	0.623	-2.472	1.482
Paediatrics	-1.060	0.684	-0.048	-1.549	0.122	-2.403	0.283
Emergency	0.178	0.776	0.007	0.229	0.819	-1.346	1.702
ICU	-2.073	0.665	-0.099	-3.117	**0.002**	-3.379	-0.768
Other	0.289	0.581	0.016	0.497	0.620	-0.853	1.430
Years of work experience	0.095	0.493	0.010	0.192	0.847	-0.873	1.063
Professional title	-0.214	0.446	-0.021	-0.480	0.632	-1.089	0.661
Work intensity	-0.602	0.282	-0.065	-2.133	**0.033**	-1.156	-0.048
Children	-0.409	0.470	-0.031	-0.871	0.384	-1.332	0.513
SC1	1.536	0.452	0.18	3.401	**0.001**	0.649	2.422
SC2	1.084	0.448	0.123	2.420	**0.016**	0.205	1.963
SC3	1.220	0.473	0.134	2.579	**0.010**	0.291	2.148
SC4	1.327	0.411	0.163	3.226	**0.001**	0.520	2.135

*R* = 0.581, *R*^*2*^ = 0.338, adjusted *R*^*2*^ = 0.326, *F* = 27.937, *P* < 0.001. SC1-SC4, items pertaining to the spiritual climate

## Discussion

This study investigated the level of psychological empowerment among Chinese nurses as well as how the spiritual climate affects nurses’ psychological empowerment.

Our study revealed that nurses’ PES score was (45.49 ± 6.42), thus indicating a moderate to high level; this value is slightly greater than the values reported in several recent studies [34–36]. The reason for this difference may pertain to the fact that the study conducted by Zhao et al. focused on nurses from the emergency department and that emergency nursing leads nurses to exhibit low levels of psychological empowerment due to its time-sensitivity, criticality, complexity, and other characteristics [34]. Second, the percentage of participants in this study who had a bachelor’s degree or higher level of education (83.5%) was higher than the levels reported in previous studies [35, 36], and a prior study revealed that a higher level of education is associated with more psychological empowerment [18]. Nevertheless, nurses’ PES scores were reported to be significantly higher in the study conducted by Arshadi et al. [37]. The reason for these different results may be related to the different time points of data collection. The survey used in this study was conducted after the COVID-19 pandemic, whereas the sample investigated by Arshadi et al. was recruited during the course of COVID-19, which was associated with a sharp increase in the number of ICU patients in the hospital. Due to the corresponding shortage of manpower, nurses were required to take ownership of their work and to make decisions concerning their work style more frequently, thereby enhancing nurses’ job self-efficacy to some extent; thus, their level of psychological empowerment was likely to be higher. Moreover, among the four dimensions of PES, the job impact dimension was associated with the lowest score (8.24 ± 3.00); job impact refers to the extent to which individuals can influence the outcomes of their organizations in terms of strategy, administration, management, and operations. Nurses usually have little influence on healthcare policy [38], and societal stereotypes of nursing focus mainly on caring for patients and the dominance of doctors in overall healthcare activities, thus weakening nurses’ influence on the sector and organizations. In contrast, the dimension associated with the highest score was job competence (12.68 ± 1.67), thus indicating that nurses have all the skills necessary to perform their jobs as well as the ability and confidence required to do so. Psychological empowerment is a motivational structure that positively influences individual behaviour [39]. Therefore, managers should pay attention to the current status of psychological empowerment among nurses and develop practical training programs to improve the state of such psychological empowerment.

A review of the literature on nurses’ psychological empowerment revealed that several sociodemographic factors influence psychological empowerment [17]. In our study, the results of a one-way ANOVA revealed that age, department, years of work experience, professional title, work intensity, and children (yes/no) have statistically significant effects on the psychological empowerment scores; namely, older nurses, those working in the ICU, those with more years of work experience, those with higher professional titles, those facing lower work intensity, and those with children exhibit relatively high psychological empowerment scores. One possible explanation for this finding is that as age and years of work experience increase, nurses’ work experience and sense of “ownership” in the hospital are strengthened, and they can independently control the decision to complete their work and perceive the significance of the work [40]; in addition, the higher a nurse’s professional title is, the more opportunities that nurse has to participate in decision-making [41], thus causing the nurse to feel as if he or she can influence the development of the unit and the department. However, a study conducted by Ibrahim et al. [42] indicated that age and years of work experience had no statistically significant effect on nurses’ psychological empowerment, and psychological empowerment was reported to be higher among married nurses, nurses with master’s degrees, and formal nurses in the study conducted by Khoshmehr et al. [18]. The reasons for these differences may be the different allocations of nursing human resources and work assignments in other countries.

*Pearson’s* correlation analysis revealed significant positive correlations between spiritual climate and psychological empowerment as well as its four dimensions; accordingly, the better the nurses’ perceived spiritual climate was, the higher the level of psychological empowerment they exhibited, a finding which is in line with the results of previous studies on the relationships among the organizational climate, a healthy work environment and psychological empowerment [43, 44]. Susuki et al. [45] surveyed half-time nurses working in a Japanese hospital and found that a positive and diverse climate in the workplace allowed these nurses to express their ideas about working, which was necessary for enhancing their psychological empowerment. A spiritual climate is an appropriate work environment in addition to the climates mentioned in the study highlighted above. Nurses working in a unit that provides them with encouragement and support are motivated to commit themselves to their work and experience the meaning of their job more fully. Soliman et al. [12] argued that a spiritual climate involves not only the individual’s relationship with colleagues but also the respect felt by the individual in this climate; that is, everyone in the department can express ideas, and everyone respects and understands each other when such a spiritual climate is present within the workplace, thereby enhancing nurses’ job competence and having a positive impact on psychological empowerment. This finding suggests that managers should attach importance to the spiritual climate in the work environment and implement relevant management strategies to establish a positive climate with the goal of enhancing nurses’ job competence and impact.

Multiple linear regression analyses revealed that with regard to the general information of nurses, psychological empowerment differed significantly only with regard to nurses working in the ICU and work intensity. Simultaneously, age, years of work experience, professional title, and children (yes/no) were statistically significant according to the one-way ANOVA; these variables were not included in the regression equation model. Compared with nurses working in other departments, the psychological empowerment scores exhibited by ICU nurses were the lowest in this study, possibly because of the critical and complex condition of patients in the ICU as well as the heavy, intense, and difficult nature of the corresponding nursing tasks [46]. Psychological empowerment enables the individual to perceive a relationship with the work environment, and a poor work environment can inhibit the flow and expression of nurses’ spiritual needs [20], thereby affecting their psychological empowerment. An impressive body of literature has highlighted the prevalence of moral distress among ICU nurses [47]. Moral distress is associated with low psychological empowerment [48], and it provides a basis for understanding the low level of psychological empowerment exhibited by ICU nurses; hence, that nursing managers should pay special attention to the psychological empowerment of ICU nurses and take action to change the corresponding management model, involve nurses in therapeutic decision-making for patients, and improve the level of psychological empowerment exhibited by ICU nurses. In addition, our study showed that work intensity is a negative predictor of psychological empowerment among nurses, such that nurses who face high levels of work intensity exhibit lower levels of psychological empowerment and vice versa. Nursing work is professionally demanding and risky since it is associated with multiple simultaneous sources of work stress. Maslach theorized that burnout is a state and that excessive workload is a core cause of burnout [49], such that the experience of burnout negatively affects work competence and self-efficacy. In contrast, nurses who face low levels of work intensity exhibit higher levels of job satisfaction [50], thus stimulating their intrinsic motivation to work and enhancing the perceived meaning of their work. Nursing managers should thus implement a balanced allocation of nursing human resources and rationalize the work intensity of nurses in light of the needs of patients’ conditions and nurses’ workload. Furthermore, the 4 items pertaining to the spiritual climate are all predictors of psychological empowerment among nurses, which, alongside nurses working in the ICU and work intensity, can explain 32.6% of the variance in nurses’ psychological empowerment. A subtle spiritual climate links the individual to his or her work when colleagues listen to, accept, and respect the individual’s opinions and ideas, thereby enhancing the individual’s perception of the meaning and purpose of his or her work and enabling that individual to achieve self-growth and progress [51]. A spiritual climate can enable individuals to experience a sense of belonging and identity when they share their inner experiences with colleagues, thereby reducing the negative aspects of work and increasing individuals’ confidence, competence, and impact on their work [13]. In a positive working climate, nurses can express themselves and cope with stress effectively, thus leading to more stable psychological empowerment at work. The findings of this study provide a basis for efforts to enhance the psychological empowerment of nurses, revealing that establishing a good spiritual climate in the workplace is beneficial in this context.

This study has some limitations that must be taken into account. First, this study focused solely on nurses from five hospitals in Sichuan Province, thus giving rise to geographical limitations. Hence, the findings should be replicated more widely, such as in other provinces. Furthermore, we use convenience sampling, which, although simple and easy to use, may result in selection bias. Finally, this study’s assessment of psychological empowerment was based on self-assessment, the results of which are subjective, which may lead to potential bias in terms of reporting. Further research is needed to explore other influencing factors and mechanisms associated with nurses’ psychological empowerment as well as intervention strategies that can be used to improve the psychological empowerment of nurses.

## Conclusions

The findings of this study showed that the level of psychological empowerment exhibited by nurses working in five hospitals in Sichuan Province, Southwest China, was at only a moderately high level and still requires improvement. The spiritual climate was positively correlated with psychological empowerment. Multiple linear regression analysis revealed that working in the ICU, work intensity, and the 4 items pertaining to the spiritual climate influenced nurses’ psychological empowerment, explaining 32.6% of the total variance. This finding suggests that nursing managers should pay more attention to ICU nurses, allocate human resources scientifically in the field of nursing, arrange the nursing workload reasonably, establish a harmonious working climate, improve nurses’ psychological empowerment, mobilize their work motivation, and provide patients with high-quality nursing services.

## Data Availability

The datasets used and analyzed during the current study are available from the corresponding author upon reasonable request.

## Abbreviations

LSE: Least squares method
PES: Psychological Empowerment Scale
C-SCS: Chinese version of the Spiritual Climate Scale
SD: Standard deviation
ANOVA: Analysis of variance

## References

[1] Tai C, Chen D, Zhang Y, Teng Y, Li X, Ma C (2024). Exploring the influencing factors of patient safety competency of clinical nurses: a cross-sectional study based on latent profile analysis. BMC Nurs.

[2] WHO. State of the world’s nursing report 2020: executive summaries. https://www.who.int/publications/i/item/9789240003279

[3] Foà C, Bertuol M, Baronchelli E, Beltrami G, Toninelli S, Zamboni L, Artioli G (2021). The influence of media representations on citizens’ perceptions towards nurses: a comparison between before and after the COVID-19 pandemic. Atenei Parmensis.

[4] Ansah Ofei AM, Paarima Y, Barnes T, Kwashie AA (2021). Staffing the unit with nurses: the role of nurse managers. J Health Organ Manag.

[5] Yang JS, Hao DJ (2018). Dilemmas for nurses in China. Lancet (London England).

[6] Haahr A, Norlyk A, Martinsen B, Dreyer P (2020). Nurses experiences of ethical dilemmas: a review. Nurs Ethics.

[7] Aloweni F, Ayre TC, Teo I, Tan HK, Lim SH (2022). A year after COVID-19: its impact on nurses’ psychological well-being. J Nurs Manage.

[8] Friend ML, Sieloff CL (2018). Empowerment in nursing literature: an update and look to the future. Nurs Sci Q.

[9] Malak MZ, Abu Safieh AM (2022). Association between work-related psychological empowerment and quality of nursing care among critical care nurses. J Nurs Manage.

[10] Wang S, Liu Y (2015). Impact of professional nursing practice environment and psychological empowerment on nurses’ work engagement: test of structural equation modelling. J Nurs Manage.

[11] Doram K, Chadwick W, Bokovoy J, Profit J, Sexton JD, Sexton JB (2017). Got spirit? The spiritual climate scale, psychometric properties, benchmarking data and future directions. BMC Health Serv Res.

[12] Soliman M, Di Virgilio F, Figueiredo R, Sousa MJ (2021). The impact of workplace spirituality on lecturers’ attitudes in tourism and hospitality higher education institutions. Tour Manag Perspect.

[13] Spreitzer GM (1995). Psychological empowerment in the workplace - dimensions, measurement, and validation. Acad Manage J.

[14] Li H, Shi Y, Li Y, Xing Z, Wang S, Ying J, Zhang M, Sun J (2018). Relationship between nurse psychological empowerment and job satisfaction: a systematic review and meta-analysis. J Adv Nurs.

[15] Saleh MO, Eshah NF, Rayan AH (2022). Empowerment predicting nurses’ work motivation and occupational mental health. Sage Open Nurs.

[16] Şenol Çelik S, Sariköse S, Çelik Y (2023). Structural and psychological empowerment and burnout among nurses: a systematic review and meta-analysis. Int Nurs Rev.

[17] Sha M, Sun DH (2023). Research progress on the status quo and influencing factors of nurses’ psychological empowerment. Mod Nurse.

[18] Khoshmehr Z, Barkhordari-Sharifabad M, Nasiriani K, Fallahzadeh H (2020). Moral courage and psychological empowerment among nurses. BMC Nurs.

[19] Ahmad N, Oranye NO (2010). Empowerment, job satisfaction and organizational commitment: a comparative analysis of nurses working in Malaysia and England. J Nurs Manage.

[20] Cruz JP, Alquwez N, Albaqawi HM, Alharbi SM, Moreno-Lacalle RC (2018). Nurses’ perceived spiritual climate of a hospital in Saudi Arabia. Int Nurs Rev.

[21] Zhang Y, Wu X, Wan X, Hayter M, Wu J, Li S, Hu Y, Yuan Y, Liu Y, Cao C (2019). Relationship between burnout and intention to leave amongst clinical nurses: the role of spiritual climate. J Nurs Manage.

[22] Garg N, Mahipalan M, Poulose S, Burgess J (2022). Does gratitude ensure workplace happiness among university teachers? Examining the role of social and psychological capital and spiritual climate. Front Psychol.

[23] Bella RLF, Quelhas OLG, Ferraz FT, Barboza DV, França SLB (2021). An initial approach to increase job satisfaction through workplace spirituality. Front Psychol.

[24] Cruz JP, Alquwez N, Balay-Odao E (2022). Work engagement of nurses and the influence of spiritual climate of hospitals: a cross-sectional study. J Nurs Manage.

[25] Fradelos E, Alexandropoulou CA, Kontopoulou L, Papathanasiou IV, Tzavella F (2022). Factors affecting Greek nurses’ caring behaviors: the role of nurses’ spirituality and the spiritual climate of hospitals. J Relig Health.

[26] Jufrizen, Sari M, Nasution MI, Radiman, Wahyuni SF (2019). The strategy of spiritual leadership: the role of spiritual survival, workplace spirituality and organizational commitment at private universities. Bus Social Sci.

[27] Wu X, Hayter M, Lee AJ, Yuan Y, Li S, Bi Y, Zhang L, Cao C, Gong W, Zhang Y (2020). Positive spiritual climate supports transformational leadership as means to reduce nursing burnout and intent to leave. J Nurs Manage.

[28] Mok E, Au-Yeung B (2002). Relationship between organizational climate and empowerment of nurses in Hong Kong. J Nurs Manage.

[29] El-Salam GA, Ibrahim MM, Mohsen MM, Hassanein SE (2008). Relationship between organizational climate and empowerment of nurses in Menoufiya hospitals, Egypt. East Mediterr Health J.

[30] Thomas KW, Velthouse BA (1990). Cognitive elements of empowerment - an interpretive model of intrinsic task motivation. Acad Manage Rev.

[31] Norman G, Monteiro S, Salama S (2012). Sample size calculations: should the emperor’s clothes be off the peg or made to measure?. BMJ (Clinical Res ed).

[32] Sun N, Li QJ, Lv DM, Lin P, Lu GZ, An XM (2011). The psychometric properties of the Chinese version of the Problem areas in psychological empowerment scale (PES): scale development. J Clin Nurs.

[33] Wu XX, Zhang Y, Wu JF, Wan XJ, Hu Y, Liu YB, Gong WJ (2019). Study on reliability and validity of the Chinese version of the spiritual ClimateScale. Chin Nurs Res.

[34] Zhao FF, Wang J, Zhang ML, Xu KY (2021). Effects of professional identity and psychological empowerment on job burnout and implicit nursing knowledge sharing among emergency nurses. Nurs J Chin PLA.

[35] Akkoç İ, Türe A, Arun K, Okun O (2022). Mediator effects of psychological empowerment between ethical climate and innovative culture on performance and innovation in nurses. J Nurs Manage.

[36] Zhang S, Liu Y, Li G, Zhang Z, Fa T (2022). Chinese nurses’ innovation capacity: the influence of inclusive leadership, empowering leadership and psychological empowerment. J Nurs Manage.

[37] Arshadi Bostanabad M, Namdar Areshtanab H, Shabanloei R, Hosseinzadeh M, Hogan U, Brittain AC, Pourmahmood A (2022). Clinical competency and psychological empowerment among ICU nurses caring for COVID-19 patients: a cross-sectional survey study. J Nurs Manage.

[38] Van Der Cingel M, Brouwer J (2021). What makes a nurse today? A debate on the nursing professional identity and its need for change. Nurs Philos.

[39] Silen M, Skytt B, Engstrom M (2019). Relationships between structural and psychological empowerment, mediated by person-centred processes and thriving for nursing home staff. Geriatr Nurs.

[40] Shi XH, Dou C, Gao YF, Xu H, Zhou XY, Leng M (2021). A study on the current status and correlation between psychological empowerment and coping styles of newly recruited nurses. Chin Gener Prac Nurs.

[41] Sarıköse S, Çelik S. Structural and psychological empowerment among newly graduated nurses and related factors: a mixed methods study. J Adv Nurs 2023. 10.1111/jan.16022 38062629

[42] Ibrahim M, Sayed HY (2014). Nurse’s psychological empowerment and perceived autonomy in university and teaching hospitals at Menofia Governorate/Egypt. J Nurs Educ Pract.

[43] Trus M, Galdikiene N, Balciunas S, Green P, Helminen M, Suominen T (2019). Connection between organizational culture and climate and empowerment: the perspective of nurse managers. Nurs Health Sci.

[44] Zeng J, Guo SB, Zheng QX, Liu XW, Lin HM, Hu AF, Yang Y, Wei BR (2023). The mediating effect of psychological empowerment on the relationship between work environment and clinical decision-making among midwives: a multicentre cross-sectional study. BMC Nurs.

[45] Susuki T, Kida R, Takemura Y, Ichikawa N, Kunie K, Koyanagi H (2022). Work-related communication mediates the relationship between perceived diversity climate and psychological empowerment among part-time nurses: a cross-sectional study. J Nurs Manage.

[46] Asadi N, Salmani F, Asgari N, Salmani M (2022). Alarm fatigue and moral distress in ICU nurses in COVID-19 pandemic. BMC Nurs.

[47] Carnevale FA (2020). Moral distress in the ICU: it’s time to do something about it!. Minerva Anestesiol.

[48] Lamiani G, Borghi L, Argentero P (2017). When healthcare professionals cannot do the right thing: a systematic review of moral distress and its correlates. J Health Psychol.

[49] Maslach C, Schaufeli WB, Leiter MP (2001). Job burnout. Annu Rev Psychol.

[50] Niskala J, Kanste O, Tomietto M, Miettunen J, Tuomikoski AM, Kyngäs H, Mikkonen K (2020). Interventions to improve nurses’ job satisfaction: a systematic review and meta-analysis. J Adv Nurs.

[51] Ashmos DP, Duchon D (2000). Spirituality at work: a conceptualization and measure. J Manag Inq.

